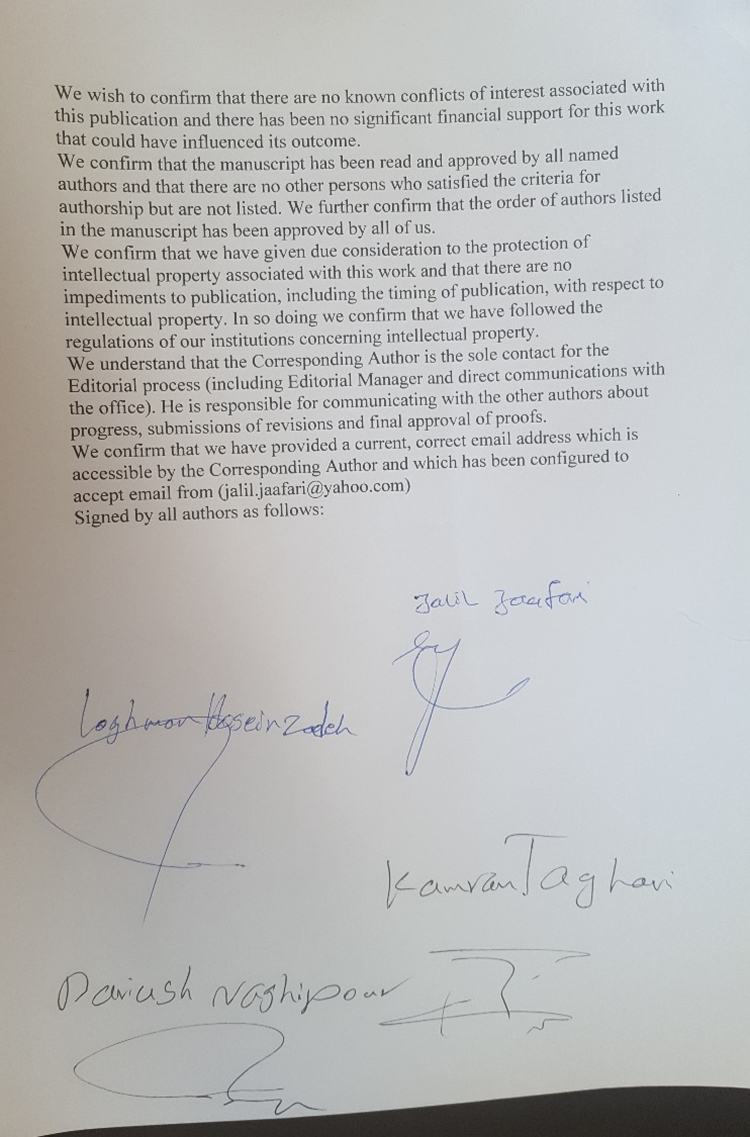# Characterization, kinetic, thermodynamic and isotherm data for diclofenac removal from aqueous solution by activated carbon derived from pine tree

**DOI:** 10.1016/j.dib.2018.03.068

**Published:** 2018-03-22

**Authors:** Dariush Naghipour, Loghman Hoseinzadeh, Kamran Taghavi, Jalil Jaafari

**Affiliations:** a School of Public Health, Guilan University of Medical Sciences, Rasht, Iran; b Department of Environmental health engineering, School of Public Health, Tehran University of medical sciences, Tehran, Iran

**Keywords:** Adsorption, Diclofenac removal, Kinetic, Thermodynamic, Isotherm data

## Abstract

The usage of low cost material as adsorbent would be admirable from environmental point of view. Thus, herein, this data set present a simple method for providing an adsorbent from activated carbon derived from pine tree. The prepared adsorbent was applied to remove diclofenac from aqueous solution. The characterization data of the adsorbent was analyzed using FTIR method. The FTIR test results showed that adsorbent has a functional group that is useful in adsorption process. It was conducted in laboratory scale and the adsorption technique was batch technique. The information regarding isotherms of diclofenac adsorption were listed. The Langmuir isotherm was suitable for correlation of equilibrium data with correlation coefficient value of 0.999. Adsorption of diclofenac by adsorbent from activated carbon follows pseudo second order model with correlation coefficient value (*R*^2^) of 0.9997. The data implied that the maximum adsorption capacity of adsorbent to uptake diclofenac from aqueous solution was obtained 54.67 mg/g. The acquired data indicated that the adsorption of diclofenac by the adsorbent prepared from activated carbon derived from pine tree is a promising technique for treating diclofenac bearing wastewaters.

## Specifications Table

**Table:** 

Subject area	Chemical Engineering
More specific subject area	Adsorption process
Type of data	Table, image, figure
How data was acquired	The uptake of diclofenac by the carbon nanotube as adsorbent (qe) was determined based on the subtraction of the initial and final concentration of adsorbate using a series of batch tests in a shaker- incubator instrument.
	Diclofenac concentration measurement was performed by spectrophotometer in 292 nm (Shimadzu, DR5000)
	Fourier transform infrared (FTIR) spectroscopy, was used for determine the characteristics of the adsorbent.
Data format	Analyzed
Experimental factors	The adsorbent of activated carbon derived from pine tree was prepared from heated in 400 °C for 2 h and activated in 800 °C for 2 h by N_2_ gas.
	Data of activated carbon derived from pine tree were acquired for diclofenac removal from aqueous solution
Experimental features	The adsorbent of activated carbon derived from pine tree for diclofenac adsorption from aqueous solution.
Data source location	Guilan University of medical sciences, Rasht, Iran
Data accessibility	Data are accessible with the article

## Value of the data

• The synthesized adsorbent has great potential application in related of pollutants removal from aqueous solution.
• Information of this data article including, isotherm, kinetic, and thermodynamic parameters will be informative for modeling and predicting the adsorption capacity and mechanism of diclofenac removal by activated carbon.
• The acquired data will be advantageous for the scientific community wanting to scale up and design an adsorption column with adsorbent of activated carbon as medium for the removal of diclofenac containing waters or wastewaters.

## 1 Data

**Fig. 1:**
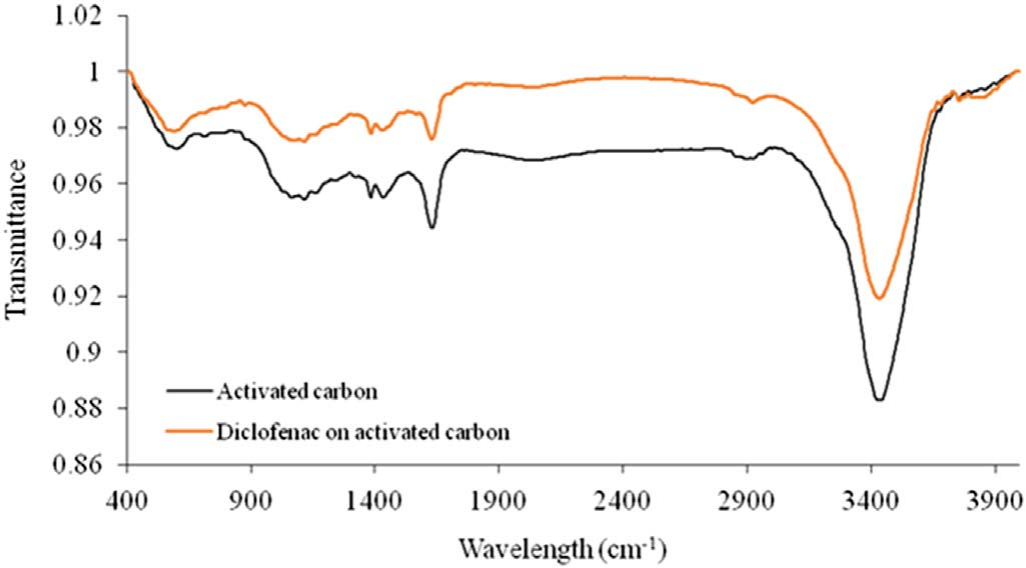
FTIR spectra of activated carbon derived from pine tree before and after of diclofenac adsorption.

The FTIR for the activated carbon adsorbent before and after adsorption at wave numbers from 400 to 4000 cm^−1^ were given in Fig. 1. The kinetics, isotherms, and thermodynamic parameters were estimated using models listed in Table 1. The data of isotherms, thermodynamic and kinetics for adsorption of diclofenac onto activated carbon is presented in Table 2, Table 3.

**Table 1:** Kinetic and isotherm model/equations used in this data article.

Model	Functional form	Plotting	Constants
Pseudo first order	$\ln(1-\frac{q_{t}}{q_{e}})=-k_{1}*t$	$\ln\;\frac{q_{t}}{q_{e}}Vs$ t	*q_e_* is the amounts of the adsorbed substance (mg/g) at equilibrium
			*q_t_* is the amounts of the adsorbed substance (mg/g) at t time
			
Pseudo second order	$\frac{t}{q_{t}}=\frac{1}{k_{2}q_{e}^{2}}+\frac{1}{q_{e}}*t$	*t*/*q_e_ Vst*	*K*_1_ is the constant of speed (L/min)
			*k*_2_ is the constant of speed (g/mg min)
			
Langmuir	$\frac{C_{e}}{q_{e}}=\frac{1}{q_{m}.K}+\frac{C_{e}}{q_{m}}$	$\frac{C_{e}}{q_{e}}\mathrm{Vs}\;C_{e}$	*q_e_* is the adsorbed amount of diclofenac per unit weight of adsorbent at equilibrium (mg/g)
			*C_e_* is the equilibrium concentration of the diclofenac (mg/L)
			
Freundlich	$\log\;q_{e}=\log\;k_{F}+\frac{1}{n}\;\log\;C_{e}$	$\log\;q_{e}\;\mathrm{Vs}\;\log\;C_{e}$	*q_m_* (mg/g) is the maximum theoretical diclofenac capacity
			*K* (L/mg) is Langmuir constant related to the affinity of binding sites
			
Temkin	$q_{e}=B_{1}\;\ln\left(k_{t}\right)+B_{1}\;\ln\left(C_{e}\right)$	$q_{e}\mathrm{Vs}\;\ln\;\mathrm{Ce}$	The *k_t_* is the Temkin isotherm constant (L/g)
			The *B* is the heat of sorption (J/mol)
			
Thermodynamic equations	Δ*G*° = − *RT* ln *K_Th_*; Δ*G*° = Δ*H*° − *T*Δ*S*°; ln *K_T_* = (Δ*S*°/*R*) − (Δ*H*°/*RT*)	ln *K*_*t*_*Vs* 1/*T*	*R* is universal gas constant (8.314 J mol/K)
			T is the absolute temperature in °K
			Δ*G*° is the Gibb's free energy change
			Δ*H*° is the enthalpy change
			Δ*S*° is the entropy change

**Table 2:** Isotherm and thermodynamic data for diclofenac adsorbed onto the adsorbent from activated carbon.

Parameter	Value
Langmuir	
*q_m_* (mg/g)	54.67
*K_L_* (L/mg)	0.84
*R*^2^	0.999
	
Freundlich	
*n*	8.2
*K_f_* (mg/g)	34.8
*R*^2^	0.95
	
Temkin	
*K_T_* (J/mol)	561.1
*b* (J/mol)	5.4
*R*^2^	0.95
	
Thermodynamic parameters (283.15 °K)	
(kJ/mol) Δ*G*	− 3.4
(kJ/mol) Δ*H*°	6.2
(J/mol K) Δ*S*°	34.79
pH_ZPC_	8.2

**Table 3:** Kinetics data for diclofenac adsorbed onto the activated carbon.

Parameter	Value
*q_e,exp_* (mg/g)	62.44
Pseudo first order	
*q_e_* (mg/g)	22.96795538
*k*_1_ (min^−1^)	0.0313
*R*^2^	0.8614
	
Pseudo second order	
q_e_ (mg/g)	64
*k*_2_ (g/mg min)	0.0035
*R*^2^	0.9997

## 2 Experimental design, materials and methods

### 2.1 Materials

#### 2.1.1 Carbon nanotube preparation

For preparation of carbon nanotube, the wastes branches of pine bark were gathered from the pin tree in Rasht, Iran. The collected pine bark masses was first washed extensively with running tap water for around 30 min followed by deionized water for removing debris and san and then shipped to the laboratory. Thereafter, the prepared pine bark masses were put into a muffle furnace in 400 °C for 2 h, and activated in 800 °C for 2 h by N_2_ gas. The dried activated carbon was ground to achieved a particle size of a 25-mesh. The uniformed particles of activated carbon was applied in diclofenac adsorption experiments.

### 2.2 Adsorption experiments

Adsorption of diclofenac with the adsorbent of activated carbon derived from pine tree was performed using batch adsorption technique. There are several experimental steps to determine the optimum condition of each variation. The shuffling of the sample was performed with a shaker at a speed of 150 rpm at room temperature. The water samples after shaking will be filtered using filter paper, then the sample water is tested with a Spectrophotometer (repeated 3 times). The determination of adsorption kinetic type was performed by determining the adsorption capacity of diclofenac solution on different time variations of 2, 5, 10, 15, 20, 30, 45, 60, 120, and 150 min. The determination of adsorption isotherm type was performed by determining the adsorption capacity of diclofenac solution on different concentration variations of 50, 100, 200, 300 and 400 mg/L. Adsorbent is used according to the optimum dose of activated carbon (0.8 g/L, optimum pH (pH7), and optimum contact time (45 min) [1], [2], [3], [4], [5], [6], [7], [8].

### 2.3 Characterization of adsorbent from activated carbon

The characterization of adsorbent from activated carbon derived from pine tree for before and after Adsorption was carried out using fourier transform infrared (FTIR). The Characterization of adsorbent activated carbon derived from pine tree was carried out using fourier transform infrared (FTIR) which aimed to analyze and to find out the functional groups of adsorbent from activated carbon derived from pine tree. The pH point of zero charge determination (pHzpc) of the activated carbons were carried out by adding 0.1 g of activated carbons to 200 mL solution of 0.1 M NaCl whose initial pH has been measured and adjusted with HCl 0.1 N or NaOH 0.1 N solutions. The containers were sealed and placed on a shaker for 24 h after which the pH was measured [9], [10], [11], [12], [13], [14], [15], [16], [17].

## 3 Data analysis

The efficiency of diclofenac adsorption by adsorbent from activated carbon is calculated according to Eq.

Adsorption efficiency

$$E=\frac{C_{e}\;-\;C_{0}}{C_{0}}\times 100$$

Where *C_o_* is initial concentration (mg/L) and *C_e_* is final concentration (mg/L).

While the adsorption capacity is calculated according to.

$$q_{e}=\frac{\left(C_{e}\;-\;C_{0}\right)V}{W}$$

Where *q_e_* is adsorption capacity per weight of the adsorbent (mg/g), *V* is volume of the solution (L), *C_o_* is initial concentration of solution (mg/L), *C_e_* is final concentration of solution (mg/L), *W* is mass of adsorbent (g) [18], [19], [20], [21].

## Figures and Tables

**Fig. S1:**